# Molecular Mechanisms of Anti-Inflammatory Phytochemicals

**DOI:** 10.3390/ijms231911016

**Published:** 2022-09-20

**Authors:** Natália Cruz-Martins

**Affiliations:** 1 Faculty of Medicine, University of Porto, 4200-319 Porto, Portugal; ncmartins@med.up.pt or ncmartins@ipsn.cespu.pt; 2 Institute for Research and Innovation in Health (i3S), University of Porto, 4200-319 Porto, Portugal; 3 Institute of Research and Advanced Training in Health Sciences and Technologies (CESPU), Rua Central de Gandra, 1317, 4585-116 Gandra, Portugal; 4 TOXRUN—Toxicology Research Unit, University Institute of Health Sciences, CESPU, CRL, 4585-116 Gandra, Portugal

Naturally occurring bioactives, also known as phytochemicals, have been widely recognized and researched owing to their multiple potentialities. Used since ancient times by indigenous people for medicinal, cosmetic, culinary and formulation purposes, in recent years, there have been intense research, on the one hand, to specifically address the chemical components responsible for their ancestrally recognized potentialities, and on the other hand, to clarify their mechanisms of action with the ultimate goal of using them as an upcoming source of safer and more effective drugs than the currently available ones. In this sense, the areas of phytochemistry and phytopharmacology have suffered a marked expansion owing to this revived interest in using naturally occurring bioactives for medicinal and pharmaceutical purposes. Among their multiple potentialities, the anti-inflammatory potential of phytochemicals has been the subject of intense investigations, on the one hand, due to the various metabolic pathways they interact with, and on the other hand, due to the exponential increase in the incidence of chronic diseases. Thus, the clarification of the mechanism of action of naturally occurring bioactives and their anti-inflammatory potentialities under different inflammatory conditions is now considered a hot topic.

The inflammatory process is part of the normal functioning of the living organisms, featured by immune and non-immune cells’ activation, which act as an effective defensive system against infection and as a way to boost tissue repair. However, when the inflammatory process overcomes the levels considered normal, there is a trigger for the onset of many disorders, with chronic disorders being the most frequent. These diseases, considered as characteristic of modern societies, include, among others, osteoarthritis, cancer, diabetes mellitus, respiratory, cardiovascular, neurodegenerative, and autoimmune diseases. Owing to the economic burden they cause, intense research has been presented in this field, with a plethora of molecules being searched with the intent of developing effective drugs capable of preventing, managing and treating them. Among the multitude of molecules searched, those derived from Mother Nature have been intensively explored, given that they constitute a wide library of biomolecules, with diverse structures and cellular targets, and thus with a plethora of therapeutic opportunities. More interestingly, recently published studies have increasingly reported the ability of phytochemicals to counteract the most crucial steps in the pathogenesis of various inflammatory conditions. Taken together, these recent findings constitute the rationale for the current Special Issue.

In this Special Issue, five papers were published—four original articles and one review article. The first original work, published by Han and colleagues [1], investigated the anti-inflammatory potential of three triterpenes isolated from *Hippophae rhamnoides* L., oleanolic (OA), asiatic (AA), and maslinic (MA) acids in RAW264.7 cells with lipopolysaccharide (LPS)-induced inflammation. As the main findings, the authors stated that the three studied triterpenes, at the same doses, were capable of inhibiting the production of pro-inflammatory mediators, such as nitric oxide (NO), inducible nitric oxide synthase (iNOS), and interleukin (IL-6), with AA and MA also being capable of triggering the production of the anti-inflammatory factor, IL-10. Equally noteworthy is that the three triterpenes studied were able to inhibit the nuclear translocation of nuclear factor kappa B (NF-kB) induced by LPS, to boost the production of reactive oxygen species (ROS) and to counteract the depletion of mitochondrial membrane potential induced by LPS. In addition, the OA and AA were able to block ERK1/2, P38, and JNK1/2 phosphorylation, and OA was effective in increasing Nrf2 expression and decreasing Keap1 expression in RAW264.7 cells [1]. The second work, published by Le and coworkers [2], addressed the anti-inflammatory potential of 2 *Salix alba*-derived methanol extracts in the context of a SARS-CoV-2 peptide challenge in Caco-2/HT29-MTX co-culture, and compared the observed effect with that of acetylsalicylic acid (ASA). As the main findings, both the Salix extracts and ASA were capable of suppressing the prostaglandin E2 (PGE2) production in a dose-dependent fashion. The inhibition of cyclooxygenase 2 (COX-2) activity was also observed for ASA and one of the Salix-derived methanol extracts, while both extracts were able to suppress the production of relevant cytokines, as is the case of IL-6, IL-1β, and IL-10 [2]. In the third work, by Lee and colleagues [3], the ability of lycopene to counteract the effects of ethanol and the fatty acid palmitoleic acid (EtOH/POA) in ROS production and pancreatic acinar cell injury, namely on zymogen activation and IL-6 expression, were addressed. Lycopene revealed a great potential to counteract mitochondrial dysfunction, zymogen activation, and IL-6 expression in pancreatic acinar cells by suppressing NADPH oxidase [3]. In the fourth work, published by Chang and colleagues [4], the polymethoxyflavanoid 5-*O*-demethylnobiletin (5-DN) was addressed in vitro and in vivo for its hepatoprotective capabilities. The most remarkable findings were stated in vivo in BALB/c mice, after treatment with carbon tetrachloride (CCl4) by intraperitoneal injection to trigger acute liver damage. Among other effects, 5-DN was capable of attenuating the increase in the serum aminotransferase (AST)/alanine aminotransferase (ALT) ratio and improved liver damage, through regulating the production of ROS and downregulating the expression of CYP2E1. Similarly, it strengthened the activity of antioxidant enzymes and Bcl-2 expression, and reduced Bax, Bid, cleaved caspase 3 and 9, and apaf-1 expression [4]. In the last work, a review article written by Leite and colleagues [5], the pharmacological potential of licorice (*Glycyrrhiza glabra* L.), namely its anti-inflammatory and antioxidant effects, along with the molecular mechanisms through which the most effective compounds present in it, namely glycyrrhizin, glycyrrhetinic acid and dipotassium glycyrrhizinate, exert such effects at the gastrointestinal (GI) level were clarified.

Taken together, data published in this Special Issue highlight the real potential of phytochemicals in inflammatory conditions, namely at the hepatic, pancreatic and GI levels, for whom promising in vivo findings have been reported to date. Although in vitro studies are of the utmost interest to determine the bioactive potential of phytochemicals and their cytotoxic potential, in vivo and particularly clinical studies are of extreme relevance, first to validate the therapeutic potential of the studied molecules and to determine their safety and toxicological profiles and secondly to stablish the likelihood of using such compounds as upcoming safe and effective drugs for anti-inflammatory purposes. In this line, in the study of Leite and colleagues [5], in which the anti-inflammatory potential of licorice was reviewed in GI conditions, only five clinical studies were listed, of which only one was terminated (phase 2), three were recruiting (two phase-1 and one not available) and one had an unknown status (phase 2, 3). Interestingly, in all these listed studies, a formula containing licorice was used, being all used in the Traditional Chinese medicine system. This underlines, contrarily to what has been defended for years in the traditional system of medicine, that the use of several molecules in a single formula is more effective, safer and efficient than the use of a single component. Even in the most preliminary studies, as is the case of in vitro experiments with screening purposes, the use of plant extracts instead of single compounds reveals synergistic capabilities, as evidenced by the study of Le and coworkers [2], who showed that the methanol extracts from Salix were more effective at counteracting inflammation, as evidenced by their ability to inhibit the enzymatic activity of COX-1 and COX-2 enzymes, than the most abundant compound present in the matrices studied, 2′-*O*-acetylsalicortin, a phenolic compound belonging to the salicylates group. Additionally, noteworthy in this study is that among both the extracts studied, that with the higher total phenolic content revealed to be the most effective in a concentration-dependent manner [2].

In short, data obtained by the authors who published their works in this Special Issue underline the huge need to develop deeper pre-clinical studies in order to clarify the real contribution of minority compounds present in some matrices and how they mechanistically interact to boost the bioactive effects observed when compared to the use of a single compound. Equally important to address is the clinical efficiency of several phytochemicals-rich formulations in inflammatory conditions, and how such bioactives present in it have the ability to potentiate the pharmacological activity of several drugs, as has been reported for the use of anticancer drugs in combination with naturally occurring bioactives [6,7,8,9,10], and even through the development of nanoformulations [11].
